# The Hospital. Nursing Section

**Published:** 1904-11-26

**Authors:** 


### The Hospital.

### Nursing Section.

Contributions for this Section of "The Hospital" should be addressed to the Editor, "The Hospital," Nursing Section, 28 & 29 Southampton Street, Strand, London, W.C.

No. 948.—Vol. XXXVII. *SATURDAY, NOVEMBER* 26, 1904.

### Notes on News from the Nursing World.

### OUR CHRISTMAS DISTRIBUTION.

As an instance of the usefulness of our Christmas distribution of clothing, the following little story just told by the matron of a large London hospital will be of interest to those readers who have contributed, and intend to again contribute, to this charity. A poor woman, living in a little village in Huntingdonshire, was sent up to the hospital by her doctor in the country. It was found that it would be necessary for her to undergo a serious abdominal operation. Her only reason against being admitted as an in-patient for the operation was that there would be no one at home to tend and mend for her seven little children. She knew that their clothing could not last without her patches and darns. The matron thereupon offered to forward a new garment to each of the seven children, if she would stay to have her operation. Upon that condition the woman consented, and successfully underwent her operation. The clothes sent to the children were from The Hospital Clothing Distribution. "I cannot tell you," the matron continued, "how intensely grateful I always am for the things sent. Ours is one of the poorest and lowest districts in London. Many of our out-patients are in rags, and I can often help them from the stock of clothes sent by The Hospital, and we try to give every in-patient a garment at Christmas."

We have to acknowledge the receipt of two large parcels of clothing sent anonymously, one from Grimsby; a parcel from Policy No. 3,099 Pension Fund; and a large parcel from Nurse Cecil of Streatham, who mentions that she was assisted by an old patient, who also contributed two of the articles. All parcels should be addressed to The Editor, 28 & 29 Southampton Street, Strand, London, W.C., with "Clothing Distribution" written on the outside.

### QUEEN ALEXANDRA AND THE QUEEN'S NURSES.

At the annual meeting of the Sheffield Queen Victoria District Nursing Association last week, great enthusiasm was caused when Mr. Harold Boulton announced that he had to read to the company a message which Queen Alexandra gave him on the previous evening for that purpose. The message was to the following effect:—"Her Majesty the Queen, as patron of the Queen's Nurses, congratulates Lady Fitzwilliam and the Sheffield Queen Victoria District Nursing Association on the very satisfactory progress they have made, and offers her best wishes for their future success. Her Majesty takes the deepest interest in the work of the Queen's Nurses among the sick and suffering poor." We are sure that the interest thus manifested by her Majesty in the work at Sheffield will act as a stimulus to the officials of the association, who require £1,000 for the work next year, towards which at present £788 only has been subscribed.

### THE SUTHERLAND BENEFIT NURSING ASSOCIATION.

A question of great importance has been raised in connection with the Sutherland Benefit Nursing Association. As far back as February last the Duchess of Sutherland, in her capacity of president, wrote to Miss E. A. Stevenson, the superintendent and general secretary, who has held that position with credit and success for some years, and suggested her resignation on the ground that her Grace wished to make a change. Miss Stevenson declined to fall in with the suggestion, but subsequently offered the committee her resignation. The offer was not accepted, and the Duchess of Sutherland thereupon signified her intention of ceasing to act as president. It can easily be imagined, however, that Miss Stevenson has since found the situation unpleasant, and at the half-yearly meeting of the association this month she signified that she had determined to retire. Her decision was accepted as final, though much regret was expressed that her services would no longer be available to the association. Without entering into details which are chiefly of local interest, we think it essential to point out that the power of asking an official to resign is not vested in the president of an association, but in the committee. The point is the more important because the Duchess of Sutherland, who has now been re-elected president, proposed in June that a lady who was qualified as a sanitary inspector should be appointed superintendent in place of Miss Stevenson; while Dr. Macrae and other members of the committee insisted at a half-yearly meeting that a fully-trained nurse must be elected. We agree with them; but if the president does not, what is to happen?

### IMMATURE YOUNG PERSONS AS NURSES.

It will be seen from the report of Our Commissioner's interview with the matron of Poplar and Stepney Sick Asylum, that there has been no question of employing nurses under 21 at that institution. The Blackwall Branch Asylum, whose authorities preferred to engage a girl at an age when she is physically unable to bear the strain, is not a training school. Happily, however, the intention was frustrated. The resolute determination of the Local Government Board to refuse to sanction the employment of any young woman under 21 as a probationer in a workhouse infirmary was shown, again, the other day at a meeting of the Braintree Guardians. There were vacancies for two probationers, and the Guardians were about to elect a girl of 19, when an inspector of the Local Government Board, who happened to be at the meeting, informed them that such an appointment would not be recognised at Whitehall. In face of this statement the idea was not persisted in, but several Guardians expressed their disgust at the regulation, which, in their opinion, "shuts out young women from beginning their training at an age when they are most fitted to learn the art of nursing." It is fortunate, both for patients and also for the future of the nursing avocation, that a directly contrary opinion is entertained by the responsible authorities. As time goes on, we earnestly hope that the number of immature young persons permitted to try their hands at nursing will be considerably diminished.

### NURSES AND TOUTS.

A case of interest to nurses was heard in the City of London County Court last week. The defendant was a nurse at the Camberwell Union Infirmary, and the plaintiff a publishing company. About the end of February this year a traveller representing the company obtained admission to the infirmary, and, without waiting for the permission of the matron or the medical superintendent, proceeded to the Nurses' Home, so that he could speak to the nurses as they came from their dinner. Two of them, chiefly because they did not like the man to go away without selling anything, decided to take one volume of a publication, and offered to pay for it then and there. The offer was refused, and they were told that they must send the money when they received the book. They were asked to put down their names and address on a piece of paper. This they did, thinking that they were ordinary pieces of paper, the agreements being so folded up as to be completely hidden. One volume of the book was sent to them, and soon afterwards they received a notice telling them that the next volume would be sent. When they wrote refusing to take any more volumes they were threatened with a summons, and were informed that "they had signed an agreement to take five books." One of them decided to fight the case, and, after hearing the evidence, the judge in the City of London County Court decided in favour of the nurse, awarding her full costs. It is significant that the traveller who got the nurses to write down their names and addresses was not called as a witness. The judgment is very satisfactory, but nurses will do well to turn a deaf ear to the blandishments of touts under any circumstances, and never sign a document of any kind without carefully reading it. They should also, if possible, preserve it.

### THE UNTRAINED MATRON AND THE EAST PRESTON GUARDIANS.

Between four and five years ago the East Preston Guardians having had some trouble owing to friction between the superintendent nurse and the untrained matron at length decided to revert to their old plan of carrying on the nursing under the supervision of the latter. This retrogressive policy having confessedly proved a failure another departure was resolved upon at the last meeting of the Guardians. The chairman of the Nursing Committee, in proposing that a head nurse should be appointed, said that the arrangement would be preferable to the appointment of a superintendent nurse because the head nurse would be under the control of the Guardians, whereas the superintendent nurse would be directly responsible only to the Local Government Board, and could not be dismissed without their consent. That is one way of looking at the matter, and we have nothing to urge against the trained nurse who was subsequently selected for the post. But the East Preston Guardians will probably find themselves in trouble again at an early date. The trained head nurse is no more likely than the superintendent nurse to work harmoniously under an untrained matron. It is strange that the East Preston Guardians cannot see that there is but one satisfactory solution of the question possible.

### MATRONS AND OPERATIONS IN NURSING HOMES.

With regard to the letter of "Inquirer" in another column, there is no general rule for the matron of a nursing home to be present at operations taking place in the institution. A matron is, of course, often present at operations in a nursing home, just as in many hospitals the matron is often present in the operating-theatre. But there is no hard-and-fast rule about it. Anyone thinking of taking an appointment as matron in a nursing home should, certainly, be prepared to attend operations. In a case where there are only two nurses on duty in the nursing home, the matron's help in the operating-room is almost a necessity, since one nurse would—presumably—be with another patient, or patients.

### STRANGE ABSENCE OF DISCIPLINE.

At the last meeting of the Thurles Guardians the sister-in-charge of the Workhouse Infirmary reported that one nurse left the house without leave on three occasions, contrary to the written directions of the medical officer, and when asked for an explanation she became defiant and insolent. The sister also reported that another nurse was never ready to take up duty at an appointed hour, and when called to order she gave no satisfaction, and refused to obey. In the course of the discussion the medical officer stated that he called one day at the infirmary, and found both nurses absent, although there was a patient dying at the time. He remonstrated with them. The nurses had been requested to appear before the guardians, but it was stated they declined to attend, and an order was made severely reprimanding them, and warning them that in the event of a repetition of their conduct they would be called upon to resign. On the face of the nature of the accusations against them, they have been treated with strange leniency.

### REGULATIONS FOR PROBATIONERS AT A LANCASHIRE HOSPITAL.

The authorities of Chorley Dispensary and Rawcliffe Hospital have been advertising for a probationer. The regulations which are forwarded to applicants are not so clear as they ought to be. Candidates are requested to apply to the Sister, who takes the place of the matron. She is invested with the power to select probationers, has the option of giving them a month's notice, and apparently can instantly dismiss them in case of misconduct, neglect of duty, or wilful disobedience. According to the 12th rule, the committee grant certificates to all nurses who pass their examinations with credit. But it is not stated by whom the certificates are granted, or who examines the nurses.

There is also a regulation to the effect that a premium of ten guineas will be required to be paid at the end of the month's probation, and in the event of the engagement being terminated before the end of the first year, the committee will be the sole judges of the proportion, if any, of the premium to be returned to the probationer. As the probationer receives no salary during her first year of training, we do not see the reason for a premium of any kind, and ten guineas is ten shillings in excess of the salary given in the second year. The regulations were sent to us for criticism, and we think that they are both ill-defined and unfair to the candidates.

### THE ROYAL DEVON AND EXETER HOSPITAL.

The chairman of the Nursing Committee of the Royal Devon and Exeter Hospital has received from Mr. D'Arcy Power the report of his examination of the nurses who have been recently trained in that institution. Mr. Power testifies to the admirable manner in which they have been taught both in the theory and practice of their art. He thinks that it might be invidious to select one part more than another, but the sick-room cookery and the "made bandages" appear to him to be especially worthy of commendation. Of the eleven candidates who presented themselves for examination, he found all worthy to receive a certificate of competency, and he suggested that Nurse Brown, having obtained the highest number of marks in her examination, might be awarded a medal should the Committee see fit to grant her one. A gold medal has since been presented to her, and also to Nurse Anderson, by the President, the medals being the gift of the chairman of the Nursing Committee, Mr. S. R. Pope.

### MISSIONARY SOCIAL GATHERING.

On Thursday last week a meeting was held by permission of Mrs. Grimké, of 34 Leinster Square, Bayswater, which was attended by nurses from various London hospitals. A warm welcome was accorded, and tea was provided by the hostess, while scattered over the rooms were books, patterns for garments needed abroad, and interesting curios from China and India. The main object of the gathering was to put before fully-trained nurses the pressing need which exists in Indian and Chinese hospitals for those qualified to undertake the training of native girls, on whose services medical missionaries now chiefly depend. Dr. Longmire, late of the Gosha Hospital, Bangalore, South India, related some touching instances of her own career, and told how at times she had been called upon to perform severe surgical operations with no help but that of a six months' probationer. Dr. Charlotte Wheeler, of Quetta, North India, gave similar interesting information. It is proposed to repeat these social gatherings during the coming year.

### SUNDERLAND UNION INFIRMARY.

Three probationers who have just completed their training at the Sunderland Union Infirmary, Miss Alice M. Duncan, Miss Maud M. Hamilton, and Miss Mary Rickaby, have been awarded certificates, the percentage of marks gained by them ranging from 88 per cent. to 78 per cent. They were examined by Dr. Bertha Webb, late resident medical officer of Gateshead Workhouse, who testified to their technical knowledge as being highly satisfactory, and complimented Miss A. S. Pruett, the superintendent nurse, upon the proficiency of their practical work, which indicated the careful training they had received under her. These nurses also recently gained the L.O.S. certificate.

### BRISTOL GENERAL HOSPITAL.

An At Home was given on Friday evening last by the chairman of the Bristol General Hospital, Mr. Joseph Storrs Fry, who during the evening distributed the medals, certificates, and prizes to the nurses. The gold medal was awarded to Nurse Upton, and the silver medal and special certificate to Nurse Lane. Certificates of merit were gained by Nurses Aldridge, Beattie, Hamson, King, Pope, Sainsbury, and Wood. Prizes were gained by the second year nurses as follows :—In anatomy, Nurse Curry first, Nurse Lawrence second; in physiology, Nurse Coomber first, Nurse Skinner second; in surgical nursing, Nurse Skinner; in medical nursing, Nurse Coomber first, Nurse Lewis second. Of the first year nurses, Nurse Grandin was first in anatomy, and Nurse Walmesley second; Nurse Grandin was first in physiology, and Nurse Coldron second; in surgical nursing Nurses Coldron and Skinner were equal; in medical nursing, Nurse Armstrong was first, and Nurses Coldron and Walmesley tied for second place.

### THE MARRIAGE OF A NURSE.

The marriage of Captain Leonard Ropner, which took place on Thursday last week at St. George's Episcopalian Church, Edinburgh, is notable because of the fact that Miss Georgina Mackay, the bride, first met Captain Ropner a little over a year ago when he sustained a serious accident while he was shooting with some friends on the moors in Perthshire and which resulted in the amputation of his left leg. Miss Mackay was the nurse called in to attend the patient after he was removed to his host's shooting lodge, and subsequently she nursed him in the spring of this year when he had to undergo a minor operation. The engagement was announced last month. Mrs. Ropner is a daughter of the late Mr. Murdock Mackay, of Castleton, Caithness, and her husband is the youngest son of Sir Robert Ropner, M.P.

### THE NURSE AND THE INDIANS.

There has been an epidemic of diphtheria among the Indians at Fort Yukon. An American nurse hearing that 125 natives had contracted the disease, and that 25 cases had proved fatal, travelled immediately from Boston to Fort Yukon, including a journey of 100 miles by canoe. When she arrived, she found that there was no doctor and very little in the way of food and medicines. Her relief work was, therefore, carried on under the greatest difficulties. She persevered, however, winning the intense admiration of the natives, until she herself fell a victim to diphtheria.

### IRISH NURSES' ASSOCIATION.

On Saturday evening last a lecture was delivered at the rooms of the Irish Nurses' Association, Dublin, by Dr. William S. Haughton, on "The Value of the Roentgen Rays in Surgery and Medicine." The lecture was both interesting and instructive. The diagrams and apparatus shown illustrated the subject with great clearness. There was a large attendance of nurses, and a cordial vote of thanks was given at the end of the lecture.

### The Nursing Outlook.

"From magnanimity, all fear above;
From nobler recompense, above applause,
Which owes to man's short outlook all its charm."

### THE NURSE'S LIMIT.

Since we called attention in the spring to the case of a nurse at one of the London hospitals who was apparently expected to act as medical officer to the out-patients, there have been several somewhat similar cases related in the daily Press, until now we have a story not unnaturally headed "A Case for Investigation." It is stated that a young man was stabbed and taken to the Royal Free Hospital, where a nurse dressed the wound. The house-surgeon never saw the case, and after a few hours the patient got up and walked out of the hospital apparently without any notice being taken. He died a few days later, and at the inquest the Grand Jury said they thought it a case for investigation, and they wanted to know why the house-surgeon never saw the patient.

There are usually two explanations of these stories: either the organisation of the institution was faulty and proper medical help was not at hand, or else the nurse overstepped her limit and took upon herself undue responsibility. The first is generally the true explanation, for no amount of scandals seem to suffice to teach hospitals the duty of providing skilful and experienced officers in the casualty department. The younger the house-surgeon or house-physician the more ready is he to diagnose off-hand and to dismiss suspicious cases as mere drunkenness. But at least he is within his limits; he is not given the post of house-surgeon till he holds his qualification. The nurse has no certificate which entitles her either to diagnose cases, or to judge whether a patient is so seriously ill as to need to be received into the wards; and the public have a right to demand that cases sent to a hospital should be treated by a registered practitioner and not by a nurse.

But there comes a period in every nurse's probation when she thinks everything goes by rule of thumb and that she can safely predict the course of one case from another. It is at this period, before time has taught caution, that the nurse is apt to usurp the place of the physician; to be very dogmatic and self-satisfied, and believe herself quite capable of treating such little things as burns and cuts. But the burnt case dies from sudden collapse, and the cut which looked so simple had after all touched vital parts. The nurse cannot be too careful to keep a clear dividing line between her duties and those of the medical man, and she is culpable indeed if she rashly, and with her eyes open, grasps at responsibilities which are beyond her limit.

There is another and more difficult point which came to the fore in the case first referred to, and that is whether a nurse should accept instructions which impose upon her the duties of house-physician or house-surgeon. Can a nurse refuse to do as she is told by her official superiors? Obedience is one of the first elements in true nursing, and if the orders given her are wrong, the blame rests on the official superior and not on the nurse. But, at the same time, it behoves the nurse whilst obeying for the moment, to ascertain that the highest officials of the hospital know what is going on.

No matron or medical man of good standing would ever leave it to a nurse to decide whether a case should be received as an in-patient or out-patient; it is a burden not fitted for a nurse's shoulders. And the plea of poverty—the plea that a hospital cannot afford to have a medical man ever on the premises—is unjustifiable. If a hospital cannot exist in a state of efficiency it should at once shut its doors, for otherwise it is a fraud upon the public. Looked at from every point of view there is nothing to justify a nurse in going beyond her limit and diagnosing and treating patients. True, the nurse may act from mere thoughtlessness or the kindest motives; but even that does not exonerate her. Her training ought to teach her above all things to keep within her own province, and to recognise the seriousness of the work she has taken up, which admits of no momentary thoughtlessness, and no carelessness even in trifles. Toiling ever in the great fight against sickness and death, the nurse should come to know that in the hands of her enemies nothing is small, nothing dare be overlooked. She should aim at the utmost precision and skill with regard to her own special duties, and, knowing how difficult they are, she should be content never to go beyond them, or rush into the dangers and cares which beset the medical man. Remember it is no question as to whether women are capable of being good medical practitioners and surgeons; that old doubt has been practically answered now, and the woman-doctor has come to stay. But in the service of humanity the good nurse is desirable and honourable, whereas the half-taught practitioner, the quack, and the conceited pretender are undesirable and dishonourable. It seems necessary to give this word of warning just now, and to beg nurses for their own sakes to be very careful not to overstep the proper limits of their professional duties.

It is one of the fears of the public about the professional nurse that she is becoming too conceited about her learning, and too ready to deal with airy carelessness in matters of serious import, which should be left to medical men. We are sorry for the nurses in the cases quoted, but we hope their sorrows may prove a warning to others.

### Lectures upon the Nursing of Infectious Diseases.

By F. J. Woollacott, M.A., M.D., B.S.Oxon, D.P.H., Senior Assistant Medical Officer, Park Hospital, Metropolitan Asylums Board, Hither Green.

### LECTURE X.—DIPHTHERITIC PARALYSIS.

The chief, and certainly the most interesting, complication associated with diphtheria is paralysis, affecting one or more of the muscles. As we have seen it results from the action of the poison of the disease on the nervous system. It most frequently begins about the third week, but sometimes earlier, while its onset may be delayed as late as the end of the second month. Its duration varies considerably —from a few days to several months—the average being about five weeks. If the patient survive complete recovery takes place and no permanent weakness is left. As a general rule it is most often met with after the more severe attacks of diphtheria, although mild cases are by no means exempt. We may estimate the severity of an attack by the extent of the membrane and the other local changes, together with the degree of disturbance of the heart's action, and the amount and duration of the albuminuria. When these symptoms are well marked paralysis is very likely, in fact almost certain, to make its appearance sooner or later, though, not uncommonly, a considerable interval elapses during which the patient is comparatively well. This early period of convalescence is often deceptive, and must not put us off our guard. We should watch the patient carefully, keep him as much as possible at rest, and avoid all exertion or excitement. His food should be nourishing but easy of digestion, in order that when paralysis begins he may be in the best possible condition to meet it. The nurse should endeavour to gain his confidence, so that if later on his strength be much reduced and his life endangered, he may submit to her treatment without resentment or objection.

In the great majority of cases signs of paralysis appear first in the soft palate, which is situated at the back of the mouth between the tonsils. Its action in health is to shut off the nasal cavities from the pharynx during the swallowing of food, or the pronunciation of certain sounds. When paralysed it hangs down flaccid and motionless, and is not raised when the patient says "Ah!" The chief symptoms are the return or regurgitation of liquid food back through the nose, and the appearance of what is called the "nasal voice." The latter is most marked in loud speaking. It depends on imperfect pronunciation of the explosive consonants such as P and B, or S and the soft G, which cannot be correctly uttered owing to the escape of air through the nose. As a rule loss of sensation accompanies the loss of power, so that tickling the palate causes no discomfort or reflex. This form of paralysis is not dangerous. The food should be taken slowly, and soft solids are swallowed most easily. In the rare cases where regurgitation is troublesome, the liquids may be given by the nasal tube.

The paralysis may be confined to the palate, and not involve any other part; but, on the other hand, it frequently spreads further, so that the patient must be carefully watched. Very often the muscles of the pharynx are the next to suffer, the result being that food cannot be swallowed with safety, and shows a tendency to enter the windpipe. The first symptom is usually a fit of coughing or choking while the patient is attempting to drink. On the occurrence of either of these the meal should be stopped, and a report sent at once to the doctor. There is always great danger, unless the proper treatment be adopted, for food may enter the larynx and cause suffocation, or particles may pass down into the lungs and set up a form of pneumonia which is almost invariably fatal. The treatment is to discontinue feeding by mouth and to feed entirely by a tube passed through the nose down to the stomach. This should be done every four hours. Owing to loss of sensation in the nose and pharynx, the passage of the tube is frequently not much felt, and the patient but little inconvenienced.

Should the paralysis extend still further the respiratory muscles may suffer. The chief of these is the diaphragm, a large, thin muscle which forms the floor of the chest. As it contracts it descends, thereby enlarging the chest from above downwards and allowing the lungs to fill with air. At the same time it depresses the contents of the abdomen, so that its anterior wall is pushed forwards. When the diaphragm is paralysed the abdomen is motionless or actually sucked in during inspiration, while the movements of the chest are much more pronounced than in health. The breathing becomes difficult and laboured. The chest heaves in the efforts to draw in a satisfactory amount of air, and the lungs tend to become choked with secretion, which the patient is too weak to cough up. In these cases much benefit will often be obtained by removing the pillows and raising the foot of the bed. By so doing we drain away, to some extent at least, the secretion from the lungs and so relieve the breathing.

Associated with paralysis of the diaphragm weakness of the vocal chords is not uncommon. The voice then becomes toneless and whispering, and the cough weak and ineffectual.

The muscles of the trunk and limbs occasionally suffer, although the loss of power is rarely complete. Frequently, indeed, there seems to be loss of tone rather than definite paralysis. The patient is limp and feeble, unable to sit upright, and easily tired. If able to walk the gait is unsteady and clumsy.

Occasionally the paralysis becomes generalised, and involves nearly every part of the body at once; so that in a well-marked case the patient is almost entirely helpless. He lies in bed, hardly able to move, and breathing with the greatest difficulty. The limbs, especially the legs, are limp and feeble. The muscles of the neck and trunk may be so much affected that he cannot turn over or raise his head. Owing to the inability to swallow, saliva collects in the mouth and is constantly running away. The voice is low and whispering, the sight weakened, and sensation dulled. The pulse is sometimes surprisingly good, but usually weak and often irregular; and in the most severe cases there may be attacks of sudden cardiac failure attended with vomiting, cyanosis, and irregular sighing respiration. Incontinence of urine and fæces may occur. The mental condition too is altered, and the patient, usually a young child, becomes apathetic and listless, taking very little notice of his surroundings. With symptoms so severe as this there is of course the gravest danger, and death may take place at any time from weakness of the heart or further implication of the respiratory muscles. But though critical, such cases are by no means hopeless, and with skilful and painstaking nursing a considerable proportion will recover.

Let us now consider what our treatment should be. We can do little or nothing to directly strengthen the muscles or remove the paralysis, but we know that if the patient lives, a perfect cure will in time result. Our efforts must therefore be directed to keeping the patient alive by means of appropriate food, and by the avoidance of any strain on the weakened muscles. He should be kept absolutely at rest and all mental excitement must be prevented, as any emotion, pleasureable or otherwise, might be followed by fatal collapse. He should not be encouraged to talk, nor should any attempt be made to "brighten" him. Toys are best withheld. Fortunately the child, as a rule, is so dull and apathetic that he is quite content to lie quiet, and has no inclination either for conversation or play. Any occasional fretfulness should be soothed at once, and a tactful nurse will soon establish a good understanding between herself and the patient. The foot of the bed should be raised, and the head turned on one side, so that the saliva and secretion from the lungs may run out of the mouth. These fluids are often very offensive, and of a brownish colour, and if necessary the mouth and throat must be swabbed out frequently. Food can never be given in the ordinary way, and even nasal feeding is so disturbing to the patient as to be dangerous, so that we must rely entirely on nutrient enemata. When changing the bedclothes, or washing the patient, every care should be taken not to interfere with him more than is absolutely necessary. By this method of treatment we are often just able to keep him alive, although life may remain at a very low ebb. In consequence of the scanty nourishment taken rapid wasting ensues, and precautions are necessary to prevent bed sores.

After a variable time, usually some days, signs of improvement appear. The breathing becomes less laboured, and the voice stronger and clearer; while fluid ceases to run out of the mouth. The child regains enough strength to raise his head, or turn over in bed. He grows more alert mentally, and begins to feel hungry and ask for food. As recovery advances the treatment may be relaxed, but always gradually and cautiously. It is a good plan, in order to test whether the power to swallow has been regained, to give a few teaspoonfuls of water. If these are taken without causing cough, milk may next be tried, and finally, after a few days more solid food.

After severe paralysis several weeks may elapse before complete recovery, and as a rule the loss of power persists longest in the legs. The patient may not be able to walk or even stand, although otherwise quite well. The nutrition of the muscles is much improved by massage, and when convalescence is established, a country or seaside holiday is very valuable.

Other varieties of diphtheritic paralysis remain to be mentioned. They are for the most part slight, cause no inconvenience and need no special treatment.

One or other of the muscles moving the eyeball may be involved, giving rise to squint; or the upper eyelid may droop and the patient be unable to raise it. This is known as ptosis.

Not infrequently there is paralysis of the ciliary muscle within the eyeball. Its function in health is to regulate and adjust the lens of the eye for near or distant vision. When it is paralysed the latter remains good, but objects close at hand cannot be clearly distinguished. The complication is a troublesome one, for the patient is unable to read unless suitable glasses are worn. Complete recovery takes place usually in about three weeks. In the case of young children ciliary paralysis may pass undetected, but it is often revealed by clumsiness in threading beads or some similar occupation.

Facial paralysis occasionally occurs. It is always slight and partial, and usually involves only the muscles about the mouth.

The skin sensation is frequently dulled, and older patients at times complain of a feeling of numbness or "pins and needles" in the feet and hands.

### The Nurses of the Poplar and Stepney Sick Asylum.

INTERVIEW WITH THE MATRON. BY OUR COMMISSIONER.

Although the huge block of buildings in Devon's Road, Bromley-by-Bow, which is known by the name of the Poplar and Stepney Sick Asylum, because it is the Poor Law infirmary for the two great East End parishes, was substantially erected thirty-three years ago, many improvements and enlargements have since been made. As I went over the institution one day last week with the matron, I noticed that a good deal still remains to be done, but the newer wards are on quite up-to-date lines; several of the old ones have been renovated, and when the theatre has been enlarged, it will be equal to all needs. The laundry is on a very extensive scale, and appears to be admirably managed. There is a pretty and well-appointed chapel, and the nurses' home, if not sufficiently commodious, is comfortable and bright.

### A Decade of Trained Nursing.

The most notable improvement at the Poplar and Stepney Sick Asylum in recent years has, however, been the introduction of trained nurses. Ten years ago, when Miss Hannaford was appointed matron, she found one trained nurse in the institution. Of course she accepted the appointment on the understanding that there was to be a revolution in the system, and in telling me the story she claimed credit for the managers who, she assures me, are keen in doing their best to make adequate provision for the patients.

"But you organised the training school?" I said.

"In conjunction with Dr. Spurrell, the medical superintendent. I came in October 1894, and he in August 1895. The single trained nurse at that date in the Asylum was the night superintendent, who was trained at the Royal Infirmary, Edinburgh."

"Were you trained at a hospital or at a Poor Law infirmary?"

"A hospital, Glasgow Western Infirmary. I remained there until I had been promoted from probationer to assistant matron. My Poor Law experience was gained subsequently at Chorlton Union Hospital, West Didsbury, Manchester, where I was matron for two years and a half. Then I came here."

"What were the different grades of the untrained nursing staff?"

"The staff was divided into head nurses, night nurse, assistant nurses, and extra nurses. The process of revolution was not violent but gradual. As vacancies took place they were filled by trained nurses, or probationers willing to undergo training. Trained sisters took the place of head nurses. Two of the old staff submitted to the new regulations, and one night nurse is still here. I found them both to be good nurses. The work of forming the school was very hard, but I had the advantage of valuable help from the assistant matrons."

### The Certificate.

"You started with the three years' training?"

"Yes, and it has since been an inflexible rule to give neither a certificate nor a testimonial under that period. The certificate is signed by the examiner, the Chairman of the Board, the medical superintendent, the Clerk to the Managers, and myself."

"At the present time all your staff are either fully trained or in course of training?"

### THE NURSES OF THE POPLAR AND STEPNEY SICK ASYLUM—*Continued.*

"They are. The number is just under a hundred. There are ten day sisters, two night sisters, eighty-three nurses and probationers in different stages of training. At the end of the three years many of the probationers become staff nurses. There are twenty-four large wards, and each sister has charge of two, four staff nurses being in charge of the other four. The number of beds is 820, and they are generally full. On Sunday, which is visiting day, upwards of a thousand people come to see their sick friends."

### Hours of Duty.

"How many nurses are on duty at night?"

"Thirty staff probationers. We do not call them staff nurses until in possession of their certificate. There are also three staff probationers on relief duty. I do not, if I can help it, put a probationer on night duty during her first year, and then I try her as one of the three on relief. There are two night superintendents in charge. During the day there are two probationers in every ward, and an extra one in the female side, where the work is always heavier, to every double ward. If a nurse falls ill, the managers allows me to have a temporary probationer, and she is put on the staff when there is a vacancy. This plan answers very well, as the temporary probationer gets an insight into the work, and we have the opportunity of judging of her capacity."

"Are the hours of duty in any way exceptional?"

"I do not think so. The staff nurses and probationers rise at 6, breakfast at 6.30, and are in the ward at 7, lunch and dress from 9.30 to 10, or 10 to 10.30, have dinner at 12.45 to 1.30, and 1.30 to 2.15, tea at 4 and 4.30, supper at 7.30 to 8. The Sisters rise at 6.30, and enter the wards at 7.30, but I need not go through their hours, which only slightly differ, except that they have an hour for dinner. The night nurses rise at 6 P.M., dine at 6.30, are in the ward at 7.30 P.M., and off at 7.30 A.M. Breakfast is at 7.45 A.M., and they are in bed at 11 A.M. They also have meals during the night."

### Leave of Absence.

"With regard to leave of absence," continued the matron, "staff nurses and probationers on day duty are off duty from 7 A.M. to 10 P.M. every third week, 2 P.M. to 10 P.M. once a week, 2 P.M. to 4.30 P.M. twice a week, 8 P.M. to 10 P.M. every evening, and 9.30 to 1.30, or 2 P.M. to 6 P.M., or 6 P.M. to 10 P.M. on Sundays. Those on night duty have a daily pass from 8.30 to 10.30 A.M., and are off duty 8.30 A.M. to 10 P.M. to next day every third week. On the first Sunday they are off duty from 8.30 A.M. to 1 P.M; on the second Sunday they attend morning service in the chapel, and on the third they have a church pass, 6 P.M. to 9 P.M. The Sisters are off from 7 A.M. to 10.30 P.M. every third week, 2 P.M. to 10 P.M. once a week, 2 P.M. to 6 P.M., and 6 P.M. to 10 P.M. once a week, 9 30 A.M. to 1.30 P.M., or 6 P.M. to 10 P.M. on Sundays, and 8 P.M. to 10 P.M. every evening. The staff nurses and probationers have 23 days' annual holiday, and the Sisters a calendar month."

"I suppose that among so many nurses you have representatives of various religions?"

"Yes; we do not make any stipulation about religion, and mass is said for the Roman Catholics in the chapel every Sunday at 7 A.M. We have two celebrations of the Holy Communion—one at 6, and another at 8.30; also morning and afternoon service. The Nonconformists have a service of their own on Thursday if they wish.

### The Age Question.

"It was recently stated that the Local Government Board had refused to sanction the appointment of a young woman of nineteen to the training staff of the Poplar and Stepney Sick Asylum. Is that correct?"

"No; the report related to the Blackwall branch of the asylum, where there are only very old people, and children. Blackwall is not a training school, and I have no responsibility for it. We receive candidates between the ages of 21 and 35.

### Salaries and Pensions.

"What is the difference in the salaries of staff nurses and probationers?"

"Probationers receive £10 the first year, £15 the second, and £20 the third. Staff nurses have from £25 to £28, increasing £1 per year. Sisters begin at £30 and rise by £1 a year to £35. We do not supply outdoor uniform, but indoor uniform is given. I suppose that some time it will be possible to pay nurses better so that they may more easily put something by for a rainy day."

"Do any of them belong to the Royal National Pension Fund?"

"Many of them are members. I belong to it myself. It is quite permissible to contract out of the Superannuation Fund. I have done so. But I strongly advise those who do to join the Pension Fund.

### The Teaching of Sick Cookery.

"A feature in your training is, I believe, the sick cookery classes?"

"This was the first Poor Law infirmary in London to start them. I was determined to have them as soon as possible, and in 1900 a teacher from the London County Council Education Board started with twelve lessons. Now there are eighteen in the year, six demonstrative and twelve practical. There is an examination at the end of the series and a certificate for sick cookery alone is given. I know that the nurses have found these classes to be of great use to them. As to the training, the matron gives a course of lectures the first year; the junior assistant matron has classes for anatomy and physiology. In the second period the medical superintendent gives a course of surgical lectures, and the senior assistant matron has classes for bandaging, splint padding, and dressing. Finally, the senior assistant medical officer gives a course of medical lectures, and the medical superintendent an advanced course of surgical lectures. Theatre classes are taken by the medical superintendent, and there is practical work in the wards taught by the Sister."

"When was the Home opened?"

"In 1896, soon after the School. There is sleeping accommodation for 28 nurses, as well as sitting-rooms for Sisters and probationers. It is not up to date, but it is very much appreciated, and is a contrast to the original state of things, when some of the night nurses had to sleep out. The library is popular, and the nurses prefer to pay a small subscription. I hope soon to have a medical library. There is an asphalte tennis court for winter, and also a lawn for croquet in summer."

"Do you get many applications for vacancies?"

"We never have to advertise for probationers. I keep a register of names of applicants, and have always sufficient on the list. An account of every applicant and of every nurse who has been here is carefully registered. The nurses get very good appointments when they leave here.

### Hospital and Infirmary Trained Nurses.

"What is your opinion about State registration?"

"I am in favour of it, and I think that it will come. I hope, meanwhile, that Poor Law infirmaries are going to be recognised as training schools for midwives. We have no lying-in wards here, but now is the time for the infirmaries to make the fight. I hope, too, that the day will come when Poor Law infirmaries will be staffed on the same lines as hospitals, and hospital-trained and infirmary-trained nurses will work together in harmony. The work here is just as good as in any general hospital. There are plenty of acute cases, and there is a great deal of nursing to be done in chronic cases. After all, it is the individual case that wants attention, and the number of cases is not a matter of moment with good nurses who take up nursing in the right spirit."

### Miss Louisa Twining.

BY A CO-WORKER.

Wednesday last week was the 84th anniversary of the birth of Miss Louisa Twining, whose work in connection with social and philanthropic questions has extended over more than half a century. The occasion was signalised by the presentation of an illuminated address, in a silver box, in acknowledgment of her public services. Miss Twining is still a worker, and takes an active interest in several movements. It was a great gratification to those who have been in various ways associated with Miss Twining in her many good works to learn a few months ago that the King had conferred upon her the Order of "Lady of Grace of the Order of the Hospital of St. John of Jerusalem in England," and thus stamped with public recognition all that she has done. Miss Twining has been so prominently connected with sickness and suffering, that a short record of what she has accomplished will, it is hoped, be acceptable to the readers of The Hospital. She has identified herself specially with the Poor Law, and her connection with the Workhouse Nursing Association, ever since its foundation, is well known; she has also taken a deep interest in district nursing and in the care of epileptics.

### Poor Law.

As early as 1847 we hear of Miss Twining's visits among the poor of Clare Market, and some years later her attention was especially directed to the Strand Workhouse, where she devoted many hours to visiting the sick, studying their conditions, and learning their needs. From this time she continued to make systematic, though informal, visits to the sick poor in workhouses, and as the years passed on, she became absorbed by other important questions, but continued to go on Sunday afternoons to the Strand Union and St. Giles's Workhouse. In 1857 was formed, mainly owing to her endeavours, the Workhouse Visiting Society, through whose investigations so much improvement has been made in workhouses. In 1861 Miss Twining gave valuable evidence before the Poor Law Commission of the House of Commons, and in 1888 evidence was again given by her before a Committee of the House of Lords. In 1861 was founded the Industrial Home for Workhouse Girls. This was the practical issue of her observation of the conspicuous flaw in workhouse management, which permitted young girls of good character to associate with the depraved. The advance in public opinion in regard to the care of the sick poor and the young in workhouses, which Miss Twining's exertions have so unceasingly promoted, resulted in 1866 in the appointment of the *Lancet* Commission to inquire into the condition and management of the pauper sick. As an outcome of the inquiry, a most important Bill was introduced by Mr. Gathorne Hardy in 1867, which gave power to separate the sick from the able-bodied paupers throughout the metropolis—the keynote of a proper system of administration. In 1879 Miss Twining aided in founding the Workhouse Infirmary Nursing Association, undertaking the important duty of honorary secretary. She resigned this position in 1882 to her able successor, Miss Wilson, but she has remained up to the present time an active and able member of the executive committee. The success of the work of this Association owes much to her sympathy, counsel, and wide knowledge. She has joined several deputations from the association to Presidents of the Local Government Board, has assisted in drawing up memorials, and has participated in special interviews with members of the Board. As recently as the summer of 1902, when the Departmental Committee on Workhouse Nursing was sitting, Miss Twining presented a valuable memorandum containing suggestions for the improvement in the administration of workhouse infirmaries and the care of the sick. It was greatly through her endeavours that women were returned as Poor-law guardians; and, from its foundation, she belonged to the Women Guardians' Society, which has recently dissolved, having effected its object. Miss Twining's own influence as a Poor-law guardian for Kensington, and later for Tunbridge Wells, was far-reaching.

### District Nursing and Care of Epileptics.

Miss Twining's deep interest in district nursing was practically shown by her starting two homes for district nurses in Kensington, and branches of the same at Worthing and Strood. In 1885 she organised in Kensington, and became honorary secretary of, a branch of the Metropolitan and National Association for Providing Nurses for the Sick Poor. She also inaugurated district nursing on a proper basis at Tunbridge Wells by the introduction of a Queen's nurse, and through her efforts a branch of the Queen Victoria Jubilee Institute was started at Rochester. Perhaps of all her works the one that touches us most is her pathetic and unselfish interest in those suffering from epilepsy. In order to secure special and satisfactory treatment for these cases she received into her own home in Queen Square, for 15 years, a certain number of epileptic patients, and she founded a home for epileptics and incurables. Up to the present moment she is still promoting this cause, and is very anxious to see suitable provision made for male patients suffering from this disease.

### General Work.

During her residence at Tunbridge Wells, Miss Twining specially urged the Boarding-out System for orphans and deserted children, and when the plan was adopted, she herself saw that suitable accommodation was provided for the first two children who were sent out. In 1857, when it was considered necessary to raise the sum of £10 000 for the enlargement of King's College Hospital, Miss Twining devoted much time and labour to help forward the movement, and, later on, she built the Twining Ward, in memory of her father, Mr. Richard Twining. Miss Twining's efforts on behalf of many societies, and on various committees, have been most valuable, and are too extensive to be enumerated. Her unflinching courage in stating her views on social questions, both at meetings and in her writings, has been of inestimable advantage.

And what words could do justice to her private life: to the deep religious motives which direct all her actions; to the quiet force of example and influence exercised day by day among those who have the honour and privilege of knowing her and of being associated with her? The simplicity of life and manner which characterise Miss Twining's striking personality are not the least factors in arousing the admiration and love of those who come intimately in contact with her, and the result of her life's work must ever live, for "she has kept her purpose with courage."

### Poor Law Matrons and State Registration.

By invitation of Miss Barton, an informal meeting of matrons of Poor Law infirmaries took place at Chelsea Infirmary on Monday.

Miss Amy Hughes, in opening the subject of State Registration as it affects Poor Law nurses, remarked that the number of nurses under the Poor Law far exceeded that of nurses in hospitals supported by voluntary contributions. She urged that State Registration, by instituting a uniform system of training, would act as a stimulus to heads of training schools. Although the bulk of surgical nursing would always be carried out by hospital-trained nurses, she claimed that in medical and chronic cases those trained under the Poor Law were equally efficient, if not superior. She combated the notion that the promoters of State Registration were aiming at class legislation, to the exclusion of the Poor Law nurses; they must be included under any scheme of the kind. What was wanted was a guarantee that the various grades of nurses knew their work thoroughly. Poor Law nurses should make themselves acquainted with the principles embodied in the two Bills which, possibly in amended form, would probably be re-introduced into Parliament, in order to be able to say in what direction amendment or alteration was desirable.

In the discussion which followed, the pros and cons of State Registration were exhaustively dealt with. It was generally agreed that the question of moral fitness was the crux of the matter, and the suggestion was made that certificates of suitability for registration should deal exclusively with technical qualities. An attempt was made to define the degree of responsibility of the matron in giving a "covering letter" with a certificate, and it was remarked that complications might arise in the suppositious case of a nurse appealing from the decision of a matron to that of a medical superintendent.

### Everybody's Opinion.

### MATRONS AND OPERATIONS IN NURSING HOMES.

"Inquirer" writes: Would any of your readers kindly say whether it be usual for the matron of a nursing home to be present at surgical operations taking place in the institution and to do the bandaging then and subsequently when there are a dozen or more nurses on the staff whom the public is told are fully trained for medical or surgical cases; of these, two nurses are on duty in the home, while the residue supply the calls to private cases?

### PRIVATE NURSING ABROAD.

"An English Nursing Sister" writes: There has been a great deal of correspondence in The Hospital about "Private Nursing," and in The Hospital of November 5th you have an article on the "Nurse Abroad," all of which I have read with great interest. May I say that I consider that private nursing abroad not only requires our best work, but it requires also the best nurses. "Up to date" "fresh from hospital" nurses are very well, but that is not enough. A private nurse abroad must have, besides patience, tact, refinement, and all the qualities you mention in your article, experience of private nursing before coming abroad. English and Americans nowadays know what good nursing is, and will only be satisfied with the best. If those who have the selection of nurses for abroad would think of this point more, perhaps the results might be better. The longer I work abroad the more I realise this point. These nurses who work abroad must, as your article says, drop all their insular prejudices, and must learn to work in harmony with others of different religions and nationalities; there must be no jealousy or friction. The care and welfare of our patient must be our first and last thought. In fact, if the modern nurse will think less of herself and more of her profession, it will be better both for herself and her profession.

### Appointments.

Infectious Diseases Hospital, Runcorn.—Miss M. A. Houghton has been appointed nurse-matron. She was trained at the Poplar and Stepney Sick Asylum, where she afterwards acted as charge nurse. She also received fever training at the Park Hospital, Hither Green, and the Grove Hospital, Tooting.

Infectious Diseases Hospital, Linacre Lane, Bootle.—Miss M. E. Deacon has been appointed sister. She was trained at the Royal City of Dublin Hospital, where she was afterwards sister.

Isolation Hospital, Wallingford.—Miss Florence May Riskin has been appointed matron. She was trained at the Marylebone Infirmary, London, and has since been superintendent nurse at the Solihull Union Infirmary, Birmingham.

Ledbury Cottage Hospital.—Miss Alice Lee Smith has been appointed matron. She was trained at the Royal South Hants Hospital, where she afterwards became charge nurse. She has since been charge nurse and matron of the Dover Hospital.

Liverpool Workhouse Infirmary.—Miss Eva Mary Edwards has been appointed charge nurse. She was trained at Burton-on-Trent General Infirmary.

Newington Workhouse, Walworth.—Miss Florence Collins has been appointed superintendent nurse. She was trained at St. Olave's Union Infirmary, where she was afterwards charge nurse and superintendent nurse. She has also been superintendent nurse at Poplar and Stepney Sick Asylum.

Royal Devon and Exeter Hospital.—Miss Alice M. Warner has been appointed night superintendent. She was trained at the Royal Devon and Exeter Hospital.

Royal Halifax Infirmary.—Miss R. B. Puxley has been appointed matron. She was trained at the Royal Halifax Infirmary, and, after being staff nurse and sister, became assistant matron, a post she has held for eight years.

St. Leonard's Hospital, Sudbury, Suffolk.—Mrs. Wolfe has been appointed matron. She was trained at the Women's Hospital, Mill Lane, Woolwich, and at the West London Hospital, Hammersmith. She has since been nurse at Greenwich Union Infirmary, district nurse at Brixham and Brecon, and sister at the Jessop Hospital for Women, Sheffield. Mrs. Wolfe holds the L.O.S. certificate.

St. Mary (Islington) Infirmary, Highgate Hill.—Miss Frances C. Wallen has been appointed assistant matron. She was trained at the Metropolitan Hospital, London, and has since held the posts of ward sister and night superintendent at Southwark Infirmary, East Dulwich, and home sister at Fulham Infirmary. She holds the L.O.S. certificate.

Union Infirmary, Lincoln.—Miss Kate Girdler has been appointed superintendent nurse. She was trained at the General Hospital, Northampton, and was afterwards charge nurse. She has since been charge and theatre nurse at Mill Road Infirmary, Liverpool, and superintendent nurse at Bedford Union Infirmary She has also done private nursing at Worcester, and district nursing at Market Harborough.

### Where to Go.

Birkbeck College, Bream's Buildings, Chancery Lane, November 26th.—Readings by Messrs. Jerome K. Jerome, Pett Ridge, and W. W. Jacobs, in aid of the Rotherhithe District Nurses' Fund.

### Echoes from the Outside World.

### Movements of Royalty.

The King and Queen, after the departure of their Portuguese Majesties from Windsor on Monday, returned to town. They were accompanied by Princess Victoria, Princess Louise, Duchess of Fife, the Duke of Fife, and by several of the royal guests who had been staying the week-end at Windsor. In the afternoon the King left town for West Dean Park, Chichester, on a visit to Mr. and Mrs. W. James. On the arrival of his Majesty at Singleton Station his host's motor-car was in waiting, and the King motored to West Dean, the avenue from the lodge gate to the house being lined by torch-bearers. He remains in Sussex until the end of the week. On Monday evening the Queen and Princess Victoria witnessed the performance of the ballet "The Entente Cordiale" at the Alhambra. On Tuesday they left London for Sandringham.

### The Visit of the King and Queen of Portugal.

Following their visit to the City on Thursday of last week, the King and Queen of Portugal expressed themselves as highly gratified with the reception given them by the people of London. On Friday the King of Portugal, with several members of the Royal Family, enjoyed good shooting at Windsor, the party being joined at luncheon in the tent on the left hand side of the Long Walk, by King Edward, Queen Alexandra, Queen Amelia, the Princess Victoria, and the Duke of Connaught, who has quite recovered from his recent accident. On Saturday there was more shooting, but the two Queens, accompanied by M. de Soveral, went out shopping and walking in Windsor. In the evening, Mr. Lewis Waller's company gave a performance of "Monsieur Beaucaire," in the Waterloo Chamber. On Sunday the King and Queen of Portugal attended the Roman Catholic Church of St. Edward, and on Monday morning, after an enthusiastic send-off by the Eton boys, their Majesties left Windsor Castle on a visit to the Duke and Duchess of Devonshire at Chatsworth. They arrived at their destination in a sharp snowstorm, and were snowed up on Tuesday.

### The Prince of Wales and the Inns of Court Mission.

On Saturday evening the Prince of Wales performed the opening ceremony at the new premises built by the Inns of Court Mission, in Drury Lane, at a cost of about £10,000. The Lord Chancellor, having welcomed his Royal Highness in the name of the Mission, and the Lord Chief Justice, having expressed the hope that his presence would inspire the junior members of the bar and law students, while they had the priceless gift of youth, to give a little of their spare time to the Mission, the Prince declared the building open. In the course of a short speech to a representative company, his Royal Highness said he was told that the erection of the five buildings comprising the institute was due, in a large degree, to the action of a small band of hard-working barristers in regular attendance at the Mission, and that, thanks to the kind assistance of lady workers, much outside help had been given in the way of obtaining funds for making it more generally known among the legal profession. He also understood that still more importance must be attached to the devoted efforts of the committee of working-men who had worked out the details and accepted the responsibility of the organisation of the institute. He declared that it was with the greatest possible pleasure that he had taken part in the ceremony, especially as it was connected with the working-man.

### The War in the Far East.

General Stoessel has telegraphed to the Tsar that Port Arthur can hold out for several months. In a despatch dated November 2nd he says that the most desperate assault was made by the Japanese on October 30th, but that they were repulsed at all points at the bayonet by the Russian Reserves and by the courage of the Volunteer scouts. The attack, he states, was not resumed on the same day, and a heap of corpses remained unburied on the field of battle. On the following day the Japanese attacked twice, but both attacks were repulsed with bayonets and hand grenades. The bombardment of the central fortress and the forts, the General says, continues incessantly. After repelling every assault—some of which were most determined—during the past nine days, the Russian troops are in the highest spirits. The Red Cross detachment is described as working with a zeal deserving of the highest gratitude, and General Stoessel asserts that the Russian surgeons are performing marvels, and that a debt of gratitude is owing to the chief huntsman of the Imperial Court for his presence in the trenches and at the most exposed surgical stations.

### Marriage by Advertisement.

The Court of Appeal heard on Friday the case of Hermann *v.* Charlesworth. The defendant appealed from a judgment of the Westminster County Court Judge, who had decided that he should return to the plaintiff, Miss Hermann, £52, the sum which she had paid him on account, as the proprietor of a matrimonial newspaper, to obtain her a husband at the end of nine months. There was an arrangement that she should pay £250 when the marriage took place. But at the end of five months, the introductions given by the defendant failing in their object, the plaintiff brought an action to recover the money she had paid him. In the County Court she gained the day, but the Court of Appeal decided against her on the ground that, as the plaintiff commenced her action when only five months out of the stipulated nine had expired, it was premature. The appeal was therefore allowed, with costs, but leave to further appeal was given.

### Losing Passengers' Luggage.

In the City of London Court on Friday a claim for £14 2s. 9d. was made against the General Steam Navigation Company for the loss of a dress-basket, containing the wearing apparel of an accountant and his wife, who went for their summer holiday by one of the company's steamers. On the return journey, the plaintiff handed a bag and dress-basket to one of the clerks at the office on Margate jetty, paying 3d. each for them. The boat by which he meant to have travelled was so crowded that he and 300 other passengers had to go by the next steamer. When he arrived at London Bridge the dress-basket was missing. The defence was that the company expressly excluded themselves from liability for any luggage, and this being set forth on the passenger ticket, judgment was given for the company, the judge suggesting that they might forego their costs.

### New Play at the Garrick Theatre.

"The Walls of Jericho," which is now running at the Garrick Theatre, contains a pungent satire on Society, and the author, Mr. Alfred Sutro, has many "straight hits" at the fashionable men and women of to-day. Jack Frobisher, the hero, is a Queensland millionaire, who has married the daughter of a scampish peer, the Marquis of Steventon. Lady Alethea plays Bridge and talks scandal immoderately, neglects her child, and indulges in a risky flirtation with an admirer. During two acts Jack Frobisher only looks on at this state of things; then he rouses himself, and does his best to set things straight and bring down the walls of Jericho. Gradually the wife is won to her husband's way of thinking, and she arranges to go with her husband and child and start on a better and nobler life the other side of the world. The play is admirably acted.

### Notes and Queries.

**FOR REGULATIONS SEE PAGE 12.**

*Nursing in Egypt.*

(55) Is it too late to try for nursing in Egypt this winter, and how should I set about getting work there?—*Nurse Caroline.*

Arrangements for nurses at the homes are made much earlier than this. You might possibly hear of a patient ordered to Egypt through an advertisement in the medical or daily papers.

*Hospital Training.*

(56) Can you kindly give me the names and addresses of general hospitals out of London where my friend and I could receive training, and where a small salary is paid from the first? It is most difficult to get into a London hospital, as we have had one year's fever training under the M.A.B. Why is this?—*E. B. F.* and *J. E. R.*

Consult the "Nursing Profession: How and Where to Train." Matrons, as a rule, prefer probationers entirely without training. It might be worth your while to apply to some of the Poor Law institutions.

*Midwifery Training.*

(57) Are there any institutions in Somersetshire or Wilts where pupils are trained in midwifery? I know of Bristol; are there any in this vicinity?—*E. E.* (Bath).

Write to the Association for Promoting the Training and Supply of Midwives, Dacre House, New Tothill Street, Westminster, London, S.W.

*Post.*

(58) Will you kindly tell me the best means of obtaining a situation as masseuse and electrician? I have had over eight years' experience in applied electricity and massage.—*Masseuse.*

It might be worth your while to join the Incorporated Society of Trained Masseuses, 12 Buckingham Street, Strand, W.C. The secretary is always ready to advise members as to the best means of obtaining work. Advertising is an alternative.

*Work.*

(59) Will you kindly tell me how I can get work? I enclose testimonials from doctor and can refer you to others. I find that the medical men here prefer nurses whom they know, and my being a stranger makes it almost impossible to get work.—*D. G.*

If you cannot start work in a place where you are known, the best advice we can give you is to join a nurses' co-operation under the management of a committee.

*Lectures.*

(60) I shall be very grateful if you would kindly tell me the best way to obtain a post as travelling lecturer on nursing and midwifery attached to a public body?—*S. A. B.*

Apply to the National Health Society, 53 Berner's Street, W., and to the London County Council, Spring Gardens, S.W.

*To Recover Fee.*

(61) I was engaged on a week's trial at 25s. and my expenses. My patient's language, under the influence of drink, was so filthy and insulting that I left the house without my box. This was forwarded and cost me 9s. 8d. Must I lose my fee and all my expenses because I went away before the end of the week?—*A. M.*

We are afraid that there is no alternative.

*Additional Training.*

(62) What advantage would it be to a male nurse to go through the "Hygiene" course at Bedford College, and obtain either the "College" certificate, or another at a public examination?—*J. L.*

It would depend on the use you wished to make of it. It would open the way to appointments relating to public health; such as lecturing on and teaching subjects comprised under that heading.

**Handbooks for Nurses.**

"The Nurses' Pronouncing Dictionary." 2s. post free.

"Surgical Ward Work and Nursing." By Dr. A. Miles. Price 3s. 6d. net; 3s. 10d. post free.

"The Human Body: Practical Physiology and Hygiene." 5s. post free.

"The Nursing Profession: How and Where to Train." 2s. net; 2s. 4d. post free.

"Preparation for Operation in Private Houses." Price 6d. post free.

The above may be obtained of all booksellers or of The Scientific Press, Limited, 28 & 29 Southampton Street, Strand, London, W.C.

### For Reading to the Sick.

TROUBLE NOT THE MASTER."

"Dead is thy daughter, trouble not the Master"—
Thus in the Ruler's ear His servants spake,
While tremblingly he urged the Saviour faster
Up the green slope from that white margined lake.

"Trouble Him not." Ah, are these words beseeming
The desolation of that awful day,
When love's vain fancies, hope's delusive dreaming
Are over, and the life has fled for aye?

Then most we need the thoughts of Resurrection,
Not the life here, 'mid pain, and sin, and woe,
But ever in the fulness of perfection
To walk with Him in robes as white as snow.

Come to us, Saviour! in our lone dejection,
Speak calmly to our wild and passionate grief,
Bring us the hopes and thoughts of Resurrection,
Bring us the comfort of a true belief.

Come! with that human voice that breaks in weeping,
Come! with that awful tenderness divine,
Come! tell us that they are not dead but sleeping,
But gone before to Thee, for they are Thine.

*C. E. Alexander.*

She is not dead but sleepeth.—*St. Matt.* ix. 24.

Thus kindly and hopefully does that kindest and most hopeful Voice that ever stirred the atmosphere of this world speak of our last change. And oh, how the very nature of death is changed when we thus think of it! Not the gloomy visitor, coming so unwelcome: but the kindly gift of our kind Saviour gently soothing us to rest. When all is said, our hearts will never be quite free from troubles, fears, anxieties, forebodings here: our feeble faith, and our many sins clouding God's face, will make sure of that: but in that last repose we shall, if we be Christ's people, sleep into forgetfulness of all these. We never shall know a real, sound, untroubled sleep in this world till that—till the weary head is laid upon the bosom of its God! "After life's fitful fever he sleeps well." How literally, how gloriously true, the great poet's words are of the true believer! Let us bless God for the pleasant thought of death which is given us by this gracious text; we need it all.—*A. K. H. B.*

As we dwell upon the memories of dear ones no more in the flesh, whose loving words were once our stay, and the graces of whose character were wont to shed a light and a gladness over our own life, let it be a sure recompense for our seeming loss to know that they are fulfilling still, in other worlds, their own characteristic ministry of love. The very virtues and graces on which we loved to dwell in this imperfect life, are ripening in the garden of the Lord, to bear their perfect fruit in the fulness of the glory of the Resurrection Day. "Such as are planted in the house of the Lord shall flourish in the Courts of the House of our God."

As we mourn the departure from among us of noble spirits—of the powerful and the wise and the saintly—of those who have been, by rare energy or a large-hearted wisdom, the builders up and the strengtheners of the spiritual Zion, great men in Israel, let us reflect for our consolation, not only that they help us still, but that they are, as we trust, carrying on, with larger opportunities, the very work of their life here, and exercising, for the glory of their Lord and to the benefit of His Church, on a grander stage, those very gifts which distinguished them while with us in the flesh.

*T. P. F. Davidson.*

